# Ultra-high-resolution computed tomography shows changes in the lungs related with airway hyperresponsiveness in a murine asthma model

**DOI:** 10.1038/s41598-021-96853-z

**Published:** 2021-09-02

**Authors:** Jae-Woo Jung, Jung Suk Oh, Boram Bae, Yoon Hae Ahn, Lucy Wooyeon Kim, Jiwoong Choi, Hye-Young Kim, Hye-Ryun Kang, Chang Hyun Lee

**Affiliations:** 1 Institute of Allergy and Clinical Immunology, Seoul National University Medical Research Center, Seoul, Korea; 2 Department of Internal Medicine, Chung-Ang University College of Medicine, Seoul, Korea; 3 Department of Radiology, Catholic University, Seoul St. Mary’s Hospital, Seoul, Korea; 4 Department of Internal Medicine, Seoul National University College of Medicine, 103 Daehak-ro, Jongno-gu, Seoul, 03080 Korea; 5 Department of Internal Medicine, University of Kansas School of Medicine, Kansas City, KS USA; 6 Department of Bioengineering, University of Kansas, Lawrence, KS USA; 7 Laboratory of Mucosal Immunology, Department of Biomedical Sciences, Seoul National University College of Medicine, Seoul, Korea; 8 Department of Radiology and Institute of Radiation, Seoul National University College of Medicine, Seoul National University Hospital, 103 Daehak-ro, Jongno-gu, Seoul, 03080 Korea

**Keywords:** Diseases, Respiratory tract diseases

## Abstract

In vivo presentation of airway hyper-responsiveness (AHR) at the different time points of the allergic reaction is not clearly understood. The purpose of this study was to investigate how AHR manifests in the airway and the lung parenchyma in vivo following exposure to different stimuli and in the early and late phases of asthma after allergen exposure. Ovalbumin (OVA)-induced allergic asthma model was established using 6-week female BALB/c mice. Enhanced pause was measured with a non-invasive method to assess AHR. The dynamic changes of the airway and lung parenchyma were evaluated with ultra-high-resolution computed tomography (128 multi-detector, 1024 × 1024 matrix) for 10 h. While the methacholine challenge showed no grossly visible changes in the proximal airway and lung parenchyma despite provoking AHR, the OVA challenge induced significant immediate changes manifesting as peribronchial ground glass opacities, consolidations, air-trapping, and paradoxical proximal airway dilatations. After resolution of immediate response, multiple episodes of AHRs occurred with paradoxical proximal airway dilatation and peripheral air-trapping in late phase over a prolonged time period in vivo. Understanding of airflow limitation based on the structural changes of asthmatic airway would be helpful to make an appropriate drug delivery strategy for the treatment of asthma.

## Introduction

Allergic asthma is characterized by its biphasic reaction pattern which consists of a type I hypersensitivity reaction that appears immediately upon allergen exposure and a delayed type IV hypersensitivity reaction that results from various cellular immune responses^1^. The early asthmatic response (EAR) presents as a rapid bronchoconstriction reaction that usually occurs within 20 min of allergen exposure and improves within a couple of hours without treatment. Allergen influx leads to mast cell degranulation by cross-linking the allergen-specific IgE molecules on their surface, which has been implicated in EAR^2^. The late asthmatic response (LAR) appears several hours after the EAR improves, and it produces a decrease in pulmonary function that can persist for 24 h and beyond^1,3^. Chemokines and cytokines synthesized and released from mast cells promote Th2 lymphocytes to migrate into the airways and play a role in the development of LARs. In addition, inflammatory mediators are released from eosinophils and neutrophils and cause pulmonary edema upon infiltration to the lungs^4–7^. Because the LAR resembles chronic asthmatic conditions, it has been used to study asthma inflammation in an experimental model^3^. However, current continuous time-series studies on both EARs and LARs are not sufficient to draw conclusions about the association between airway hyperresponsiveness (AHR) and the inflammatory responses in the lungs and airways.

AHR, a hallmark of asthma commonly used for diagnostic purposes, is the acute bronchoconstriction response that occurs in response to relatively weak stimuli that elicits little to no reactions in healthy subjects^8,9^. AHR may be induced by immunologically specific stimuli such as allergens, or immune-inert nonspecific stimuli such as methacholine, mannitol. Regardless of the individual sensitization status, the non-specific bronchoprovocation test using immune-inert nonspecific stimuli is performed to confirm the presence of AHR. Indirect bronchoprovocation testing uses agents such as cold air, mannitol, and adenosine to induce a response, while other stimuli such as histamine, leukotriene, and methacholine are used in direct bronchoprovocation testing^10^. Methacholine stimulates parasympathetic muscarinic receptors in the airway and is believed to cause a bronchoconstriction response in a way similar to that found in the EAR. The methacholine provocation test is widely used in clinical practice for its high sensitivity of 96.5%^11^. However, whether the methacholine provocation test wholly reflects the spectrum of pathophysiologic changes of the asthmatic airway caused by allergens has not been accurately validated. In addition, little is known about the similarities and differences in the mechanisms behind the bronchoconstriction response observed in the EAR to allergens and methacholine exposure^8^.

Spirometry is currently the only way to serially evaluate the airway dynamics in vivo. Imaging studies thus far have focused on revealing the static differences of airway and lung parenchyme between asthmatics and healthy patients^12–14^. As the majority of studies are human cross-sectional studies, there is a lack of information on the serial changes that occur in the airway and lung parenchyma after allergen exposure*. *In vivo studies using murine asthma models are also limited in that they do not reflect the very early changes that develop immediately upon antigen exposure. Thus, in order to better understand the airflow dynamics behind AHR, in vivo monitoring of the allergic response to noxious stimuli is needed to evaluate both the immediate and progressive changes of different parts of airway and lungs. This study successfully evaluated such changes in airway dynamics in a non-invasive manner using enhanced pause (Penh) and created a real time, anatomical representation of AHR using Ultra-high-resolution computed tomography (UHRCT).

## Methods

### Establishment of ovalbumin-induced murine asthma model

All experiments were approved and conducted by the Institutional Animal Care and Use Committee of the Institute of Laboratory Animal Resources at Seoul National University (SNU-130312-4-2). This study was carried out in compliance with the ARRIVE guidelines.

Experiments were conducted with 6-week-old female BALB/c mice (OrientBio, Seongam, South Korea) which were bred in a specific pathogen-free environment that complies with laboratory animal regulations. Five mice were assigned to both the asthma group and the control group.

**Figure 1:**
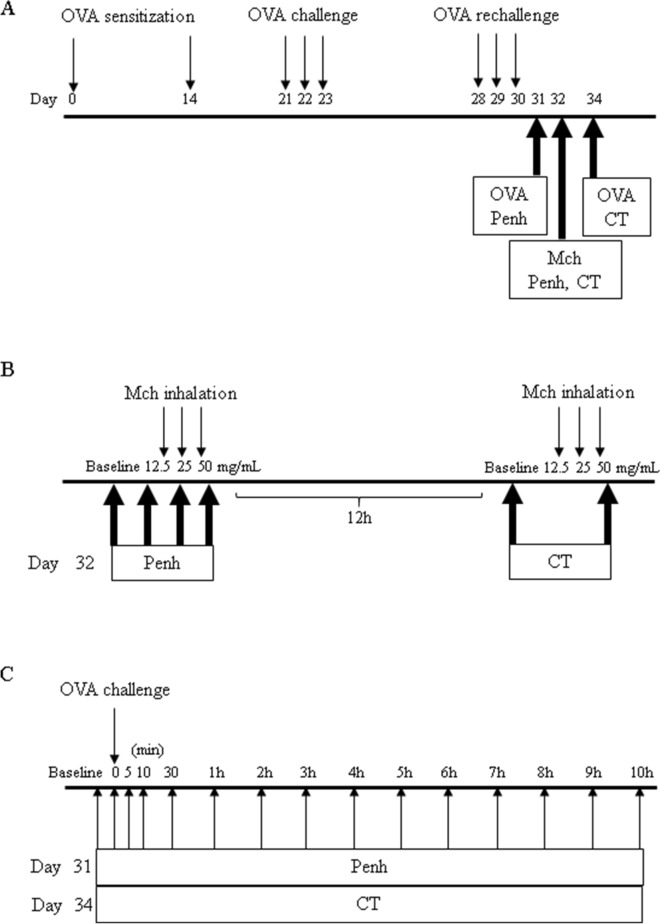
Mouse model of ovalbumin-induced asthma and study protocol. **(A)** Establishment of ovalbumin-induced asthma murine model, **(B)** noninvasive whole body plethysmography and UHRCT after methacholine challenge, **(C)** serial noninvasive whole body plethysmography and UHRCT after OVA challenge for 10 h in asthma model.

The asthma murine model was made in the following manner; 75 µg of ovalbumin (OVA) (Sigma, St. Louis, MO, USA) and 2 mg of alum (Sigma) dissolved in 200 µL of phosphate-buffered saline (PBS) was injected intraperitoneally on Days 0 and 14 to sensitize the mice. After receiving an intraperitoneal injection of ketamine (60 mg/kg), the mice were challenged with 50 µg of OVA dissolved in 20 µL of PBS as an intranasal drip on the 21st, 22nd, 23rd, 28th, 29th, and 30th days (Fig. 1A).

### Methacholine challenge and OVA challenge

The OVA challenge was conducted on the 31st and the 34th days and the methacholine challenge was conducted on the 32nd day (Fig. 1A). Anesthesia was performed with the intraperitoneal injection of ketamine (60 mg/kg) for both methacholine and OVA challenge. The mice inhaled increasing concentrations of methacholine (Sigma, St. Louis, MO, USA) for 3 min each, from 12.5 mg/ml to 25 mg/ml, and 50 mg/ml using a nebulizer (NE-U17, Omron, Kyoto, Japan). The intranasal OVA challenge was performed with a solution of 50 μg of OVA dissolved in 20 µL of PBS.

### Measurement of airway hyperresponsiveness

Penh was used as a non-invasive method to assess AHR. Penh was measured using a two-chamber whole body plethysmography (OCP3000™, Allmedicus, Anyang, Korea) for 3 min^15^.

### UHRCT for lung parenchyma and airway analysis

UHRCT images were obtained using a 128-multi detector CT scan with a 1024 × 1024 matrix (Ingenuity, Philips Healthcare, Amsterdam, Netherlands) to analyse airway and lung parenchymal changes^16,17^. Following anesthesia, UHRCT scans were performed while the mice were breathing freely. The scan parameters were as follows: 120 kvp, 175 mAs, 0.5 s rotation time, FOV 36 × 36 mm, and 0.7 mm thickness.

Two chest radiologists blinded to both the time and the provocation agent reviewed the UHRCT images. The cross-sectional inner luminal areas of the proximal Cardiac Lobe Right Main Bronchus 3 (CaRMB3) in BALB/c mice were measured using the Infinitt picture archiving and communication system (PACS, Infinitt Health Care, Maroview, Seoul, South Korea). The inner luminal area was calculated using measurements from the first third portion of each bronchus.

Lung histograms were obtained for the evaluation of the density changes of the lung parenchyma before and after allergen exposure. The density changes were measured in the following manner: after each lung was segmented at the CaRMB3 level, the Hounsfield Units (HU) of each 1 mm^2^ region of interest (ROI) was measured and the values were summed up to estimate the average densities of the lung parenchyma (Supplement 1). These serial lung histograms dynamically quantified the amount of air trapping and the extent of alveolar collapse caused by allergen exposure. Since air trapping is estimated using density threshold-based measures between inspiration and expiration, we presumed that the serial changes of density measurement at the same lung parenchyma might reflect the air-trapping or alveolar collapse. The term “ground glass opacity” is used when the mean CT attenuation is − 618.4 ± 212.2 HU, reflecting an area of hazy consolidation that is less attenuated in comparison to solid areas that have an average attenuation of − 68.1 ± 230.3 HU^18^.

### Measurement of airway and lung parenchymal changes during the early phase reaction after methacholine challenge

Under anesthesia, UHRCT scans were performed after methacholine or OVA challenge in the asthma group and the control group to evaluate the structural changes of early phase reactions to methacholine and OVA. Penh was measured after the methacholine challenge on the 32nd day, and the paired UHRCT scan was performed after 12 h on the same day (Fig. 1B). Penh was also measured after the OVA challenge on the 31st day and the paired UHRCT scan was performed on the 34th day (Fig. 1A).

### Serial measurement of AHR, airway and lung parenchymal changes after OVA challenge

The Penh measurement (day 31) and UHRCT scan (day 34) were performed repeatedly for 10 h after the intranasal OVA challenge in order to track the serial responses in the asthma group. Under anesthesia, Penh was measured at 5, 10, 30, 60, 120, 180, 240, 300, 360, 420, 480, 540 and 600 min after the intranasal OVA challenge on the 31st day. On the 34th day, the UHRCT was performed at 5, 10, 30, 60, 120, 180, 240, 300, 360, 420, 480, 540 and 600 min following the intranasal OVA challenge (Fig. 1C).

### Statistics

All data were expressed as the mean ± standard error of the mean (SEM). The paired T test was performed to confirm the airway changes before and after the methacholine or OVA challenge. Correlation analyses were performed to find associations between the lung histograms, airway diameter and Penh values. A *P* value less than 0.05 was considered to be significant.

## Results

### Airway changes during early phase reaction

**Figure 2:**
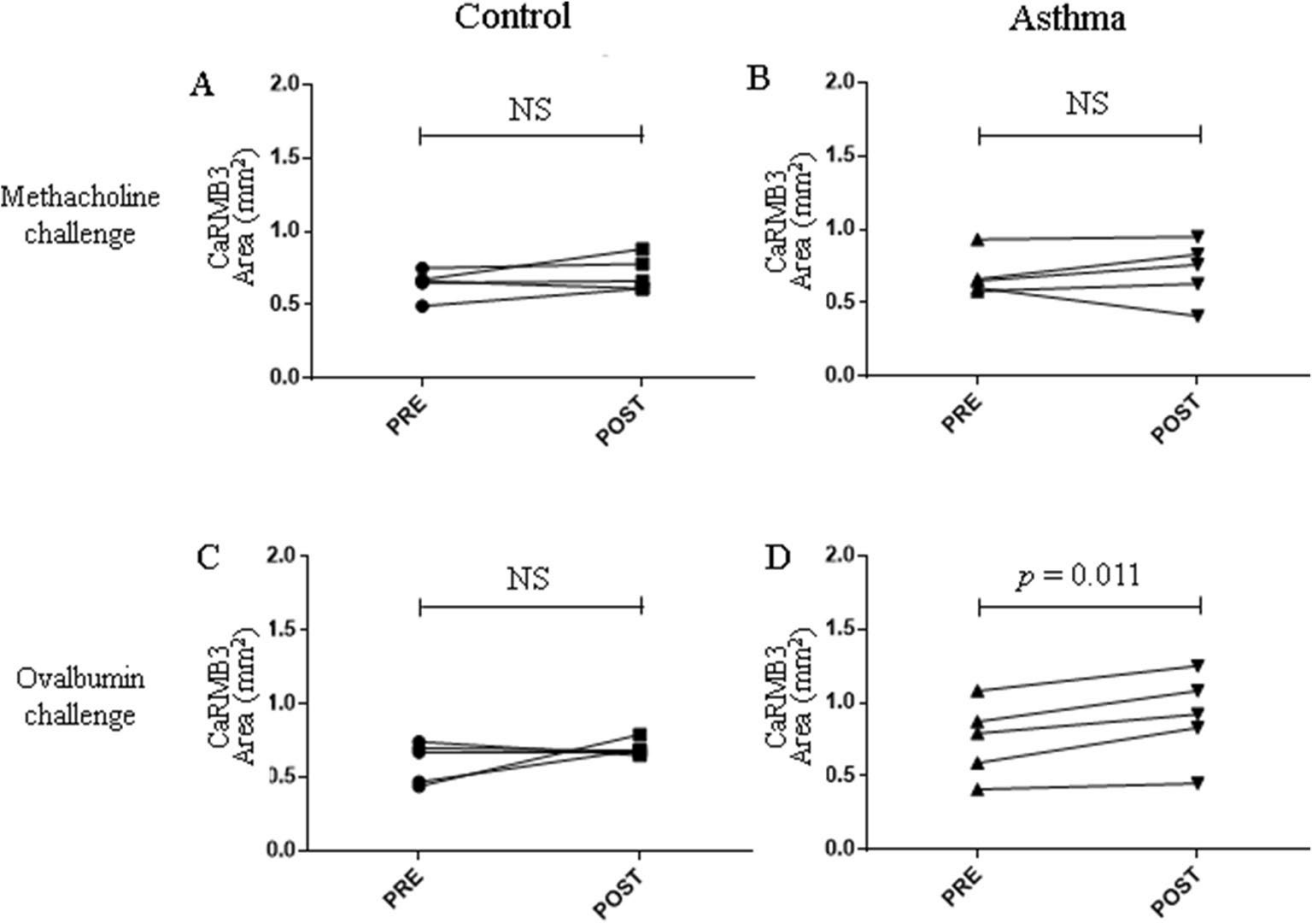
Airway changes during early phase reaction after methacholine and OVA challenge. **(A)** CaRMB3 change after methacholine challenge in control group, **(B)** CaRMB3 change after methacholine challenge in asthma group, **(C)** CaRMB3 change after OVA challenge in control group. **(D)** CaRMB3 change after OVA challenge in asthma group. In the control group, there was no change after methacholine or OVA challenge. The asthma group mice showed no significant change of airways to methacholine challenge. However, they showed slight but significant increase of airways to OVA challenge.

UHRCT was performed on BALB/c mice to compare the airway area before and after the methacholine or OVA challenge (Fig. 2). In the control group, no changes in CaRMB3 area were observed after the methacholine or OVA challenge (Fig. 2A,C). In the asthma group, there was no significant change in the CaRMB3 area after the methacholine challenge (0.684 ± 0.063 vs. 0.716 ± 0.092, *p* = 0.629, Fig. 2B) in spite of enhanced Penh (Supplement 2). In contrast, the CaRMB3 area showed a significant increase following the OVA challenge (0.748 ± 0.115 vs. 0.906 ± 0.135, *p* = 0.011, Fig. 2D).

### Parenchymal changes during early phase reaction

**Figure 3:**
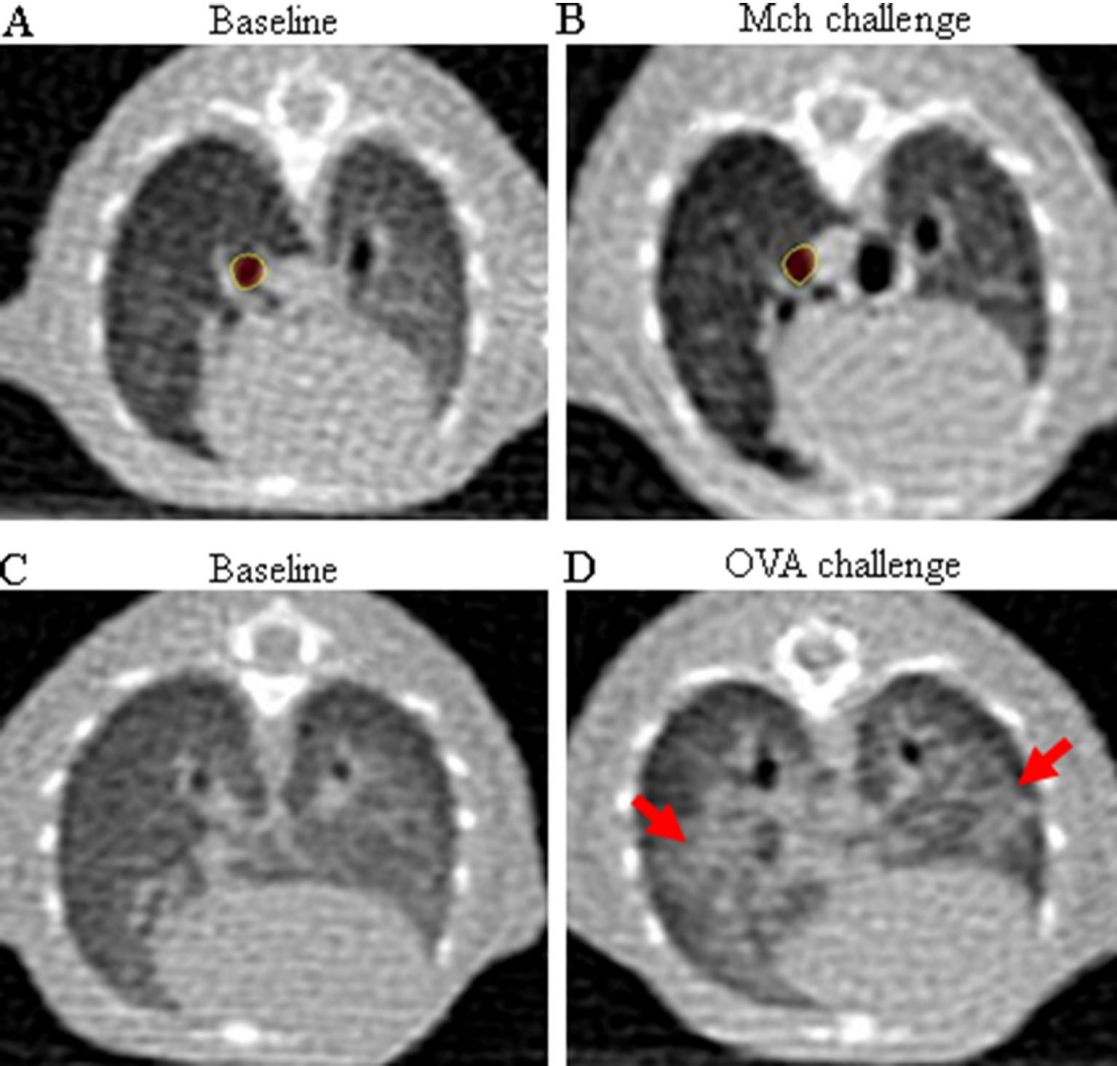
Parenchymal changes during early phase reaction in asthma model. **(A)** Baseline image before methacholine challenge. **(B)** Image of 5 min after methacholine challenge. **(C)** Baseline image before OVA challenge. **(D)** Image of 5 min after OVA challenge. Methacholine challenge did not induce any parenchymal changes nor bronchial changes. However, prominent peribronchial attenuation developed along with dilation of bronchus by OVA challenge.

In the asthma group challenged with methacholine, no immediate changes in the lung parenchyma were observed (Fig. 3A,B). However, 60% of the CT scans of the mice challenged OVA showed ground glass opacity and consolidations with an overall increase in peribronchial attenuation (Fig. 3C,D). In the control group, no parenchymal changes were observed after challenges with either methacholine or OVA (data now shown). Supplement 3 is UHRCT movies before and after OVA challenge.

### Serial changes of AHR, airway and parenchyma after OVA challenge

**Figure 4:**
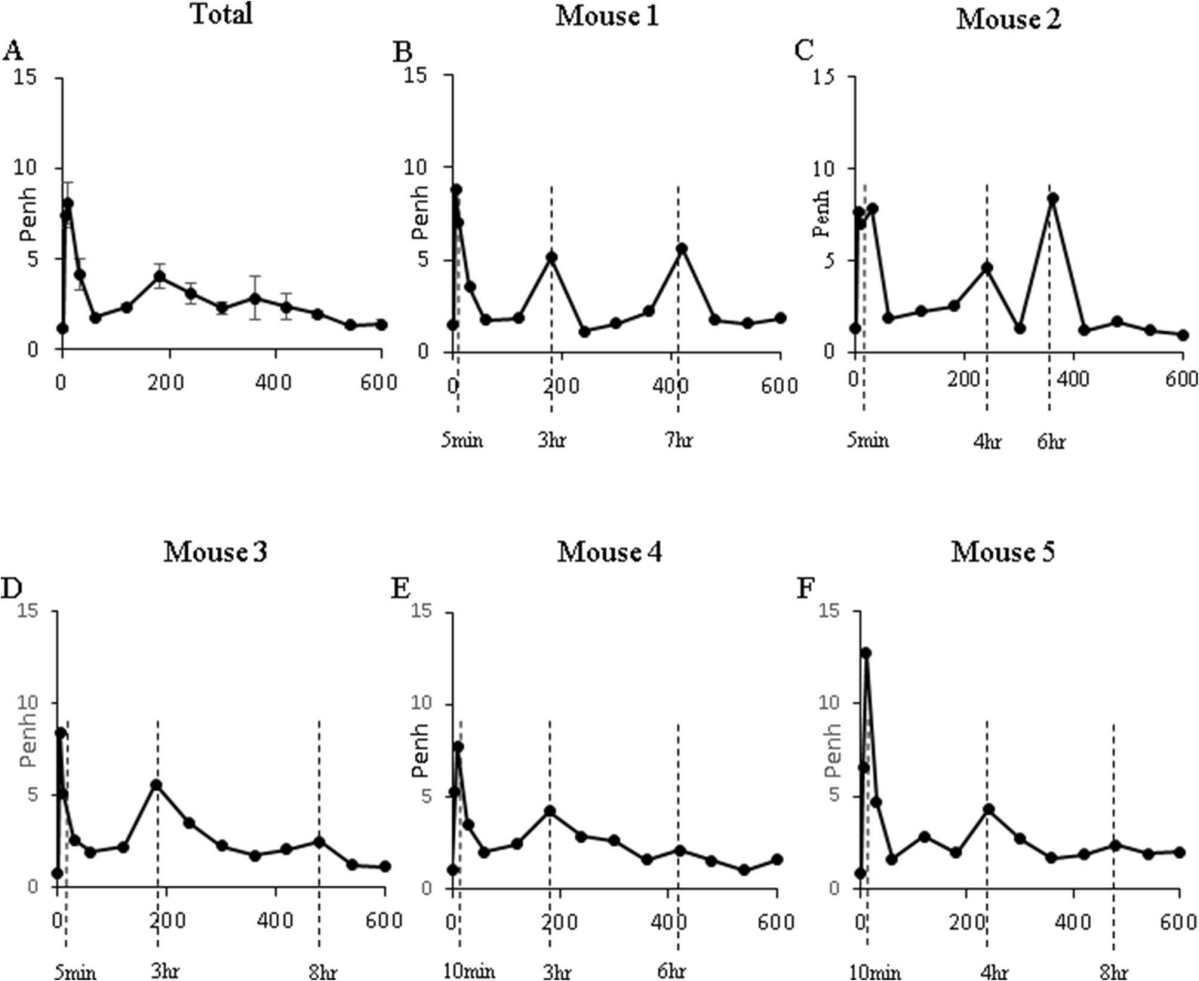
Serial changes of Penh up to 10 h after OVA challenge. **(A)** Serial mean Penh after OVA challenge. **(B–F)** Penh change in each mouse 1–5. As all together, two peaks representing EAR and LAR were observed. However, interestingly some mice showed multiple peaks of LAR.

After the OVA challenge in the asthma model, Penh was measured at 5, 10, 30, 60 min and at one-hour intervals for a total of 10 h. A rapid initial rise in Penh reflective of the EAR was observed in 5–10 min after the OVA challenge, and two LAR peaks were observed at 3 h and at 6–8 h (Fig. 4). While the first LAR peak was observed in a similar manner in all mice, the timing and degree of the second LAR was different for each individual mouse.

**Figure 5:**
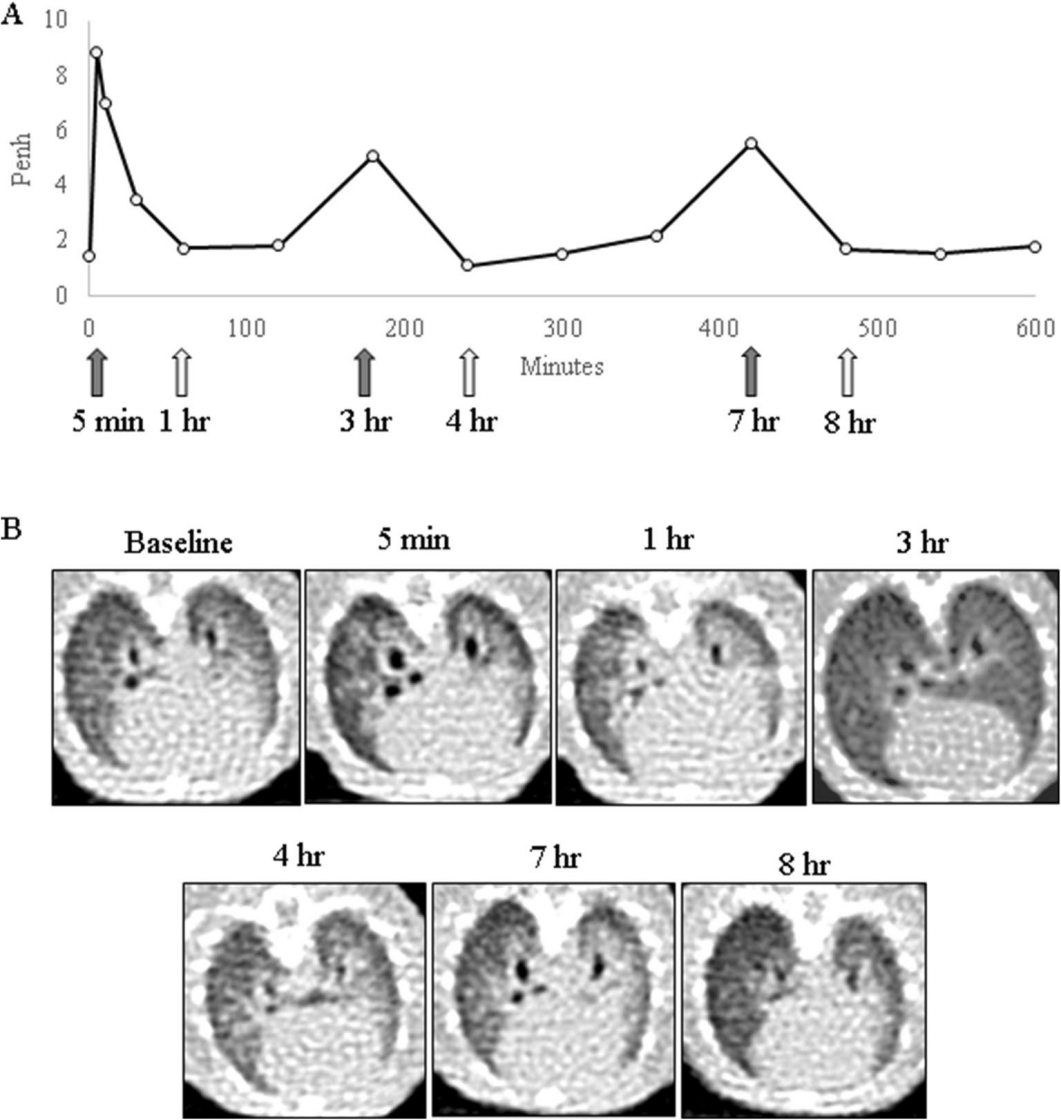
Corresponding changes of airway and Penh. **(A)** Serial changes of Penh after OVA challenge during 10 h. **(B)** Serial changes of CT imaging. When Penh increased, bronchial diameter significantly increased.

UHRCT was performed in each mouse at 5 min, 30 min, and at one-hour intervals for a total of 10 h after the OVA challenge to serially observe the structural changes (Fig. 5). When the Penh increased, the bronchial diameters of CaRMB3 showed a paradoxical increase, and when the Penh decreased to the baseline, the bronchial diameters also decreased to their original sizes.

**Figure 6:**
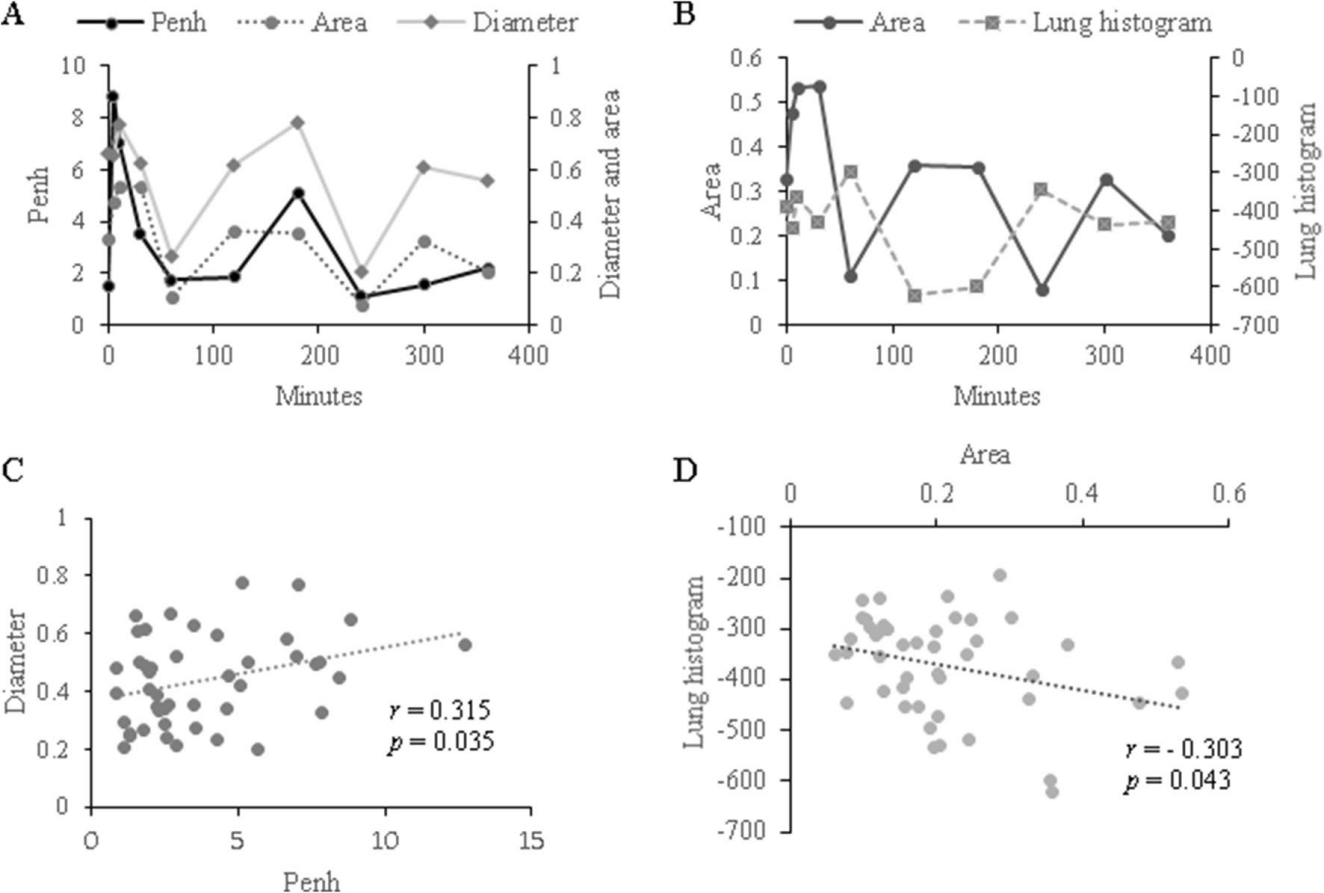
Correlation between imaging parameters. **(A)** In mouse 1, serial changes of Penh, bronchial diameter and bronchial area up to 10 h after OVA challenge. **(B)** Correlation between Penh and bronchial diameter. **(C)** In mouse 1, serial changes of bronchial area and lung histogram after OVA challenge. **(D)** Correlation between bronchial area and lung histogram. Penh was well correlated with diameter and area. Reduced airflow is correlated with wider bronchial diameter. Bronchial area was negatively correlated with lung histogram. These changes in proximal airway are also closely correlated with air trapping in periphery.

After the OVA challenge for each mouse, Penh, luminal area, airway diameter and lung histogram at each time point were measured, and correlation analyses of each index were performed (Fig. 6). The association between luminal area and Penh or peripheral air trapping (lung histogram) were compared along the time points (Fig. 6A,B). The relationship between the Penh and airway diameters showed significant positive correlation (r = 0.315, *p* = 0.035, Fig. 6C). The lung histogram, indicative of air trapping, showed a significant correlation with the narrowing of luminal area (r = − 0.303, *p* = 0.043, Fig. 6D). Together these results show that peripheral air trapping and proximal bronchial dilatation occur simultaneously in response to the airflow obstruction caused by bronchoconstriction following OVA exposure.

## Discussion

This study used imaging studies to show the paradoxical dilatation of the proximal airway and accompanying processes after allergen exposure in a murine asthma model. Our results show that the compensatory paradoxical airway dilatation occurs concurrently with peribronchial inflammation and peripheral air trapping. Additionally, the airway resistance measurement and UHRCT measurements were used to confirm the occurrence of repeated LARs for up to 10 h following OVA exposure. The structural changes of the airway and lung parenchyma that were observed in this study after allergen exposure may have a significant impact on airflow dynamics. A better understanding of the anatomical changes of airways and the ways such changes affect airflow in AHR may help us develop novel treatment strategies that focus on improving these issues.

Methacholine, a synthetic choline ester, acts as a non-selective muscarinic receptor agonist in the parasympathetic nervous system and is routinely used to assess AHR. Although the methacholine challenge test is a sensitive diagnostic tool, the differences between the allergic response caused by methacholine and by other allergens are not clearly understood. A recent study conducted in a murine asthma model reported that intravenous methacholine infusion did not consistently cause a bronchoconstriction response; instead, the airway showed heterogeneous regional responses^19^. Bayat et al.^20^ confirmed that intravenous methacholine and OVA exposure elicit fundamentally different lung responses in a rabbit asthma model. While OVA injection mainly acted on the peripheral airways and caused severe ventilation heterogeneities, methacholine evoked constriction in the central airways. This study confirmed that while OVA exposure caused a bronchoconstriction response in small airways, peripheral parenchymal changes and proximal airway dilatations, a provocation test using methacholine did not bring about the same responses. These results show that methacholine and OVA stimulation do not produce identical airway reactions; thus, methacholine stimulation cannot wholly reflect the lung parenchymal changes induced by exposures to different allergens.

In asthmatic patients, inflammatory reactions in the small airways ultimately result in airway remodelling^21,22^. Therefore, resolving inflammation in the small airways is an important goal in the treatment of asthma^23,24^. Previous imaging studies of the airway in asthma patients mainly focused on the static differences in bronchial wall thickness^12,25–28^. The ratio of the bronchial outer diameter to the inner diameter decreases in asthma, and such changes signify the hypertrophy of the bronchial wall^12,27^. This change occurs more prominently in bronchi of a relatively small diameter^13^. Meanwhile, some studies have also reported that bronchi with larger inner diameters have been observed in asthmatic patients, with the rate varying from 18 to 77%^26,27^. In this study, the changes in the small airways after OVA exposure were observed as peribronchial attenuation on UHRCT images, and the paradoxical dilatation of the CaRMB3 was perhaps the compensatory reaction to the pathologic collapse of the distal airways that were too small to be captured in our images. As reported by Wang et al., paradoxical increase in the inner diameter of the proximal bronchus was found in 52.2% of asthmatic patients and it showed an positive correlation with asthma severity^14^. These compensatory changes were successfully replicated in the murine asthma model of this study. Further studies are needed to investigate the chronic changes in airway dynamics in response to allergen exposure.

Bronchial wall thickening, ground glass opacities, consolidations and air trapping are characteristic imaging findings in asthma patients^26,29–32^. The exact air trapping was unable to measure because it was impossible to obtain inspiration and expiration images in spontaneously breathing mice. Instead, density histograms was used to quantify the degree of air trapping after allergen exposure, and the results showed that larger amounts of air trapping were associated with a greater dilatation of proximal airways in LARs. These findings can be explained by the possibility that the proximal airways dilate as a compensatory mechanism to offset the effects of peripheral air trapping caused by increased airway resistance upon allergen exposure.

The UHRCT images and the Penh of this study showed that multiple LARs occurred in all mice for up to 10 h after OVA stimulation. To the extent of our knowledge, no studies have yet confirmed the multiplicity of LARs in a murine model through serial analyses of airway resistance and lung imaging. Our findings suggest that asthma exacerbation following allergen exposure may occur repeatedly even if the allergen exposure is no longer continued, reinforcing the necessity of a long-term anti-inflammatory treatment. A recent human study identified the persistent LAR by serial monitoring the lung function and sputum cytology in asthmatics^33^. Patients enrolled in this study showed a progressive decline in pulmonary function with a simultaneous increase in sputum eosinophils after 7 h for up to 24 h after allergen exposure, indicating a persistence of the LAR^33^.

The Micro-CT is an imaging tool commonly used in small animal studies. However, the micro-CT requires a long scanning and image processing time as well as ventilatory support for the animals involved^34,35^. For the investigation of lung pathology in murine models, maintaining a resting state of spontaneous respiration through the use of anesthesia during CT scanning is required. This is the first study that used UHRCT with a 1024 × 1024 matrix instead of the conventional 512 × 512 matrix and a 36 mm FOV to obtain serial images of the airways and lung parenchyma of free breathing mice. Recently, a UHRCT capable of imaging up to 0.25 mm thickness using a photon counting CT or a 1792-channel detector and a 1024 × 1024 matrix has been developed, and this CT may be more applicable to in vivo studies of small animals^36^.

This study has some limitations. First, simultaneous performing Penh and CT were not possible. Thus, in order to produce comparable data, the Penh and CT scans were conducted with the same mice under similar conditions. Second, the Penh and CT images showed the occurrence of two or three LAR peaks within 10 h following allergen exposure, but the possibility of more LAR peaks afterward could not be ruled out although peaks that appeared later were much smaller than the first one in most mice. Therefore, a study with a longer monitoring time is required to investigate additional occurrences of LAR after the second peak. Third, the constriction of the bronchioles mainly contributes to decreases air flow during asthma attacks. As the CaRMB3 used for the measurement of luminal area and diameter belongs to the main bronchus, changes in the CaRMB3 diameter may not be reflective of the small airway changes that occur in response to allergen exposure, and the changes in the distal airways were not visible in the UHRCT scan images. Lastly, lung perfusion measurements were not performed, and information about the degree of ventilation-perfusion mismatch could not be obtained.

## Conclusion

A paradoxical increase in the diameter of proximal bronchus and distal air-trapping coupled with airflow limitation was observed on both early and late phase in murine asthma model. Understanding of airflow limitation based on the structural changes of asthmatic airway would be helpful to make an appropriate drug delivery strategy.

## Supplementary Information

Supplementary Figures.

## References

[1] Pradalier A (1993). Late-phase reaction in asthma: Basic mechanisms. Int. Arch. Allergy Immunol..

[2] Weersink EJ, Postma DS, Aalbers R, de Monchy JG (1994). Early and late asthmatic reaction after allergen challenge. Respir. Med..

[3] O'Byrne PM, Dolovich J, Hargreave FE (1987). Late asthmatic responses. Am. Rev. Respir. Dis..

[4] Broide DH (1991). Evidence of ongoing mast cell and eosinophil degranulation in symptomatic asthma airway. J. Allergy Clin. Immunol..

[5] Frew AJ (1990). T lymphocytes and eosinophils in allergen-induced late-phase asthmatic reactions in the guinea pig. Am. Rev. Respir. Dis..

[6] Walker C, Kaegi MK, Braun P, Blaser K (1991). Activated T cells and eosinophilia in bronchoalveolar lavages from subjects with asthma correlated with disease severity. J. Allergy Clin. Immunol..

[7] Gluecker T (1999). Clinical and radiologic features of pulmonary edema. Radiographics.

[8] Anderson SD (2010). Indirect challenge tests: Airway hyperresponsiveness in asthma: Its measurement and clinical significance. Chest.

[9] Chapman DG, Irvin CG (2015). Mechanisms of airway hyper-responsiveness in asthma: The past, present and yet to come. Clin. Exp. Allergy.

[10] Lee MK (2017). Nonspecific bronchoprovocation test. Tuberc. Respir. Dis. (Seoul).

[11] Yurdakul AS, Dursun B, Canbakan S, Cakaloglu A, Capan N (2005). The assessment of validity of different asthma diagnostic tools in adults. J. Asthma.

[12] Awadh N, Muller NL, Park CS, Abboud RT, FitzGerald JM (1998). Airway wall thickness in patients with near fatal asthma and control groups: Assessment with high resolution computed tomographic scanning. Thorax.

[13] Okazawa M (1996). Human airway narrowing measured using high resolution computed tomography. Am. J. Respir. Crit. Care Med..

[14] Wang D (2016). A morphologic study of the airway structure abnormalities in patients with asthma by high-resolution computed tomography. J. Thorac. Dis..

[15] Kim YK (2007). Airway exposure levels of lipopolysaccharide determine type 1 versus type 2 experimental asthma. J. Immunol..

[16] Zhu H (2017). Improved image quality and diagnostic potential using ultra-high-resolution computed tomography of the lung with small scan FOV: A prospective study. PLoS ONE.

[17] Namati E (2006). In vivo micro-CT lung imaging via a computer-controlled intermittent iso-pressure breath hold (IIBH) technique. Phys. Med. Biol..

[18] Okada T (2009). Computer-aided diagnosis of lung cancer: definition and detection of ground-glass opacity type of nodules by high-resolution computed tomography. Jpn. J. Radiol..

[19] Dubsky S (2017). Assessment of airway response distribution and paradoxical airway dilation in mice during methacholine challenge. J. Appl. Physiol..

[20] Bayat S (2009). Methacholine and ovalbumin challenges assessed by forced oscillations and synchrotron lung imaging. Am. J. Respir. Crit. Care Med..

[21] van der Wiel E, ten Hacken NH, Postma DS, van den Berge M (2013). Small-airways dysfunction associates with respiratory symptoms and clinical features of asthma: A systematic review. J. Allergy Clin. Immunol..

[22] Scichilone N (2013). Assessing and accessing the small airways; Implications for asthma management. Pulm. Pharmacol. Ther..

[23] Fujisawa T (2013). Alveolar nitric oxide concentration reflects peripheral airway obstruction in stable asthma. Respirology.

[24] Gao JM, Cai F, Peng M, Ma Y, Wang B (2013). Montelukast improves air trapping, not airway remodeling, in patients with moderate-to-severe asthma: A pilot study. Chin. Med. J. (Engl).

[25] Paik SH, Kim WK, Park JS, Park CS, Jin GY (2014). A quantitative study of airway changes on micro-CT in a mouse asthma model: comparison with histopathological findings. Allergy Asthma Immunol. Res..

[26] Park CS (1997). Airway obstruction in asthmatic and healthy individuals: Inspiratory and expiratory thin-section CT findings. Radiology.

[27] Lynch DA (1993). Uncomplicated asthma in adults: Comparison of CT appearance of the lungs in asthmatic and healthy subjects. Radiology.

[28] Gono H, Fujimoto K, Kawakami S, Kubo K (2003). Evaluation of airway wall thickness and air trapping by HRCT in asymptomatic asthma. Eur. Respir. J..

[29] Lynch DA (1998). Imaging of asthma and allergic bronchopulmonary mycosis. Radiol. Clin. N. Am..

[30] Ferrer A, Roca J, Wagner PD, Lopez FA, Rodriguez-Roisin R (1993). Airway obstruction and ventilation-perfusion relationships in acute severe asthma. Am. Rev. Respir. Dis..

[31] Kim WW (2012). Xenon-enhanced dual-energy CT of patients with asthma: Dynamic ventilation changes after methacholine and salbutamol inhalation. AJR Am. J. Roentgenol..

[32] Park SJ (2012). Quantitative analysis of dynamic airway changes after methacholine and salbutamol inhalation on xenon-enhanced chest CT. Eur. Radiol..

[33] Revez JA (2018). Sputum cytology during late-phase responses to inhalation challenge with different allergens. Allergy.

[34] Kizhakke Puliyakote AS (2016). Morphometric differences between central vs. surface acini in A/J mice using high-resolution micro-computed tomography. J. Appl. Physiol..

[35] Vasilescu DM, Knudsen L, Ochs M, Weibel ER, Hoffman EA (2012). Optimized murine lung preparation for detailed structural evaluation via micro-computed tomography. J. Appl. Physiol..

[36] Hata A (2018). Effect of matrix size on the image quality of ultra-high-resolution CT of the lung: Comparison of 512 x 512, 1024 x 1024, and 2048 x 2048. Acad. Radiol..

